# Effects of sodium bicarbonate supplementation on exercise performance: an umbrella review

**DOI:** 10.1186/s12970-021-00469-7

**Published:** 2021-11-18

**Authors:** Jozo Grgic, Ivana Grgic, Juan Del Coso, Brad J. Schoenfeld, Zeljko Pedisic

**Affiliations:** 1 Institute for Health and Sport, Victoria University, Melbourne, Australia; 2 County Hospital Schrobenhausen, Schrobenhausen, Germany; 3 Centre for Sport Studies, Rey Juan Carlos University, Fuenlabrada, Spain; 4 Department of Health Sciences, Lehman College, Bronx, NY USA

## Abstract

**Background:**

We aimed to perform an umbrella review of meta-analyses examining the effects of sodium bicarbonate supplementation on exercise performance.

**Methods:**

We systematically searched for meta-analyses that examined the effects of sodium bicarbonate supplementation on exercise performance. The methodological quality of the included reviews was evaluated using the Assessing the Methodological Quality of Systematic Reviews 2 (AMSTAR 2) checklist. Grading of Recommendations Assessment, Development, and Evaluation (GRADE) framework for downgrading the certainty in evidence was used, which included assessments of risk of bias, inconsistency, indirectness, imprecision, and publication bias.

**Results:**

Eight reviews of moderate and high methodological quality met inclusion criteria. Using the GRADE framework, evidence for the ergogenic effects of sodium bicarbonate supplementation on peak and mean power in the Wingate test and Yo-Yo test performance was classified as being of moderate quality. The evidence for these outcomes did not receive a point on the indirectness GRADE item, as “serious indirectness” was detected. Low-quality evidence was found for the ergogenic effect of sodium bicarbonate supplementation on endurance events lasting ∼45 s to 8 min, muscle endurance, and 2000-m rowing performance. Evidence for these outcomes was classified as low quality, given that risk of bias, indirectness, and publication bias were assessed as “unclear”, “serious”, and “strongly suspected”, respectively. The ergogenic effects ranged from trivial (pooled effect size: 0.09) to large (pooled effect size: 1.26). Still, for most outcomes, sodium bicarbonate elicited comparable ergogenic effects. For example, sodium bicarbonate produced similar effects on performance in endurance events lasting ∼45 s to 8 min, muscle endurance tests, and Yo-Yo test (pooled effect size range: 0.36 to 0.40). No significant differences between the effects of sodium bicarbonate and placebo were found for general mean power, muscle strength, and repeated-sprint ability.

**Conclusion:**

Based on meta-analyses of moderate to high quality, it can be concluded that sodium bicarbonate supplementation acutely enhances peak anaerobic power, anaerobic capacity, performance in endurance events lasting ∼45 s to 8 min, muscle endurance, 2000-m rowing performance, and high-intensity intermittent running. More research is needed among women to improve the generalizability of findings.

## Background

Sodium bicarbonate is a popular nutritional supplement, with studies exploring its effects on exercise performance dating back to the 1930s [1]. The effects of sodium bicarbonate supplementation have been investigated for different exercise tasks, varying in duration and intensity (e.g., high-intensity running or cycling, 200-m swimming, boxing, resistance exercise, 2000-m rowing, and repeated-sprint performance) [2–11]. However, the findings have been inconsistent, with studies reporting ergogenic, ergolytic and no significant effects [2–11]. Some of the inconsistencies between findings may be due to differences in the population analyzed, sodium bicarbonate supplementation protocols, exercise protocol, and performance outcomes. Besides these factors, the discrepancies in the findings might be due to the small sample sizes in some of the individual studies, which might have resulted in low statistical power [12]. Specifically, several studies published on this topic were performed while including only 5 to 6 participants [7–10]. One way to overcome the issue with small sample sizes in primary studies is to pool their results in a meta-analysis.

In recent years, several research groups performed meta-analyses examining the effects of sodium bicarbonate supplementation on different aspects of exercise performance [13–20]. However, meta-analyses tend to be narrow in scope. Specifically, they commonly concentrate on one specific outcome or a particular population [21]. Due to this limitation, it may be challenging to establish conclusive recommendations regarding the overall effect of sodium bicarbonate supplementation on exercise performance.

Given the increased popularity of meta-analyses, researchers have recently started to perform umbrella reviews, which endeavor to synthesize and critically evaluate information from all meta-analyses performed on a given topic [21, 22]. As several meta-analyses [13–20] explored the effects of sodium bicarbonate supplementation on exercise performance, it is timely to summarize their findings in the form of an umbrella review. Such a review is needed to: (i) evaluate the overall efficacy of sodium bicarbonate supplementation in improving exercise performance, (ii) assess the availability and quality of meta-analytic evidence, and (iii) provide recommendations for future research. Therefore, the aim of this paper was to perform an umbrella review of meta-analyses exploring the effects of sodium bicarbonate supplementation on exercise performance.

## Methods

### Search strategy

The literature search was performed across the following five databases: CINAHL, PubMed/MEDLINE, Scopus, SPORTDiscus and Web of Science. The search was performed on December 11th, 2020, using the following search syntax: (“sodium bicarbonate” OR NaHCO3) AND (exercise OR training OR muscle OR “physical performance” OR “aerobic endurance” OR “peak power” OR “mean power”) AND (meta-an* OR “systematic review”). The search was performed through the titles, abstracts and keywords of documents indexed in the databases between the database inception and the search date. The search and selection of meta-analyses were performed independently by two authors (JG and IG). Upon completion, the lists of included and excluded reviews were compared. Of note, the list of included papers was the same between these two authors.

### Inclusion criteria

The reviews that satisfied the following criteria were included in this umbrella review: (a) examined the effects of sodium bicarbonate supplementation on exercise performance in human participants, (b) analyzed the data using a meta-analysis and (c) published in English. The following criteria were outlined in the Participant-Intervention-Comparison-Outcome (PICO) process:

- *Participants:* healthy individuals, not limited to sex or age.
- *Interventions:* sodium bicarbonate supplementation.
- *Comparison group:* placebo.
- *Outcome measures:* exercise performance.

### Data extraction

Data extraction was performed following previous recommendations for umbrella reviews [23]. From each included review, we extracted the following data: (a) number of included studies, (b) pooled number of participants, (c) exercise test/outcome and (d) pooled effect sizes with their 95% confidence intervals (CI), *p*-values and *I*^*2*^*.* Data extraction was performed in duplicate by two authors (JG and IG) of the review. Data extraction files were compared between the authors and all observed differences were scrutinized and corrected. For meta-analyses that used Cohen’s *d*, the pooled effect size was classified as “small” (Cohen’s *d*: 0.20–0.49), “medium” (Cohen’s *d*: 0.50–0.79), and “large” (Cohen’s *d*: ≥ 0.80), according to Cohen [24].

### Methodological quality

We evaluated the methodological quality of the included reviews using the Assessing the Methodological Quality of Systematic Reviews 2 (AMSTAR 2) checklist [25]. We opted to use the AMSTAR 2 checklist because it is one of the most widely used instruments for the assessment of the quality of reviews and has also been previously applied in the field of sports nutrition [25, 26]. This checklist has a total of 16 items that include questions regarding the use of PICO, review registration, study inclusion criteria, comprehensiveness of the search strategy, number of authors that performed the search and data extraction, presentation of included and excluded studies, use of a scale for the evaluation of methodological quality, received funding (for authors of both primary studies and reviews), appropriateness of the meta-analysis model, mention and interpretation of heterogeneity between included studies, and investigation of publication bias. There are four possible answers in each item: “yes”, “no”, “cannot answer” and “not applicable”. “Yes” is the only answer that earns a point on a given item. Therefore, the maximum score on the checklist is 16. The quality of included reviews was classified as “low”, “moderate” or “high”, if less than 40% of items were satisfied, between 40 and 80% of items were satisfied and at least 80% of items were satisfied, respectively [26].

### Quality of evidence

Grading of Recommendations Assessment, Development, and Evaluation (GRADE) framework for downgrading the certainty in evidence was used to evaluate the quality of evidence [27]. In brief, GRADE provides a systematic method for assessing the certainty of findings in meta-analyses, and thus affords the ability to draw conclusions on the strength of practical recommendations. We used a modified GRADE scale that evaluates the risk of bias, inconsistency, indirectness, imprecision, and publication bias. Specific details on the use of this scale are reported elsewhere [26, 27]. Based on the GRADE evaluation, the quality of evidence was classified as “very low”, “low”, “moderate” or “high”. The methodological quality and quality of evidence were evaluated independently by two authors of this review (JG and IG). Upon completion, scores were compared between the authors and all observed differences were scrutinized and corrected.

## Results

### Search results

**Fig. 1:**
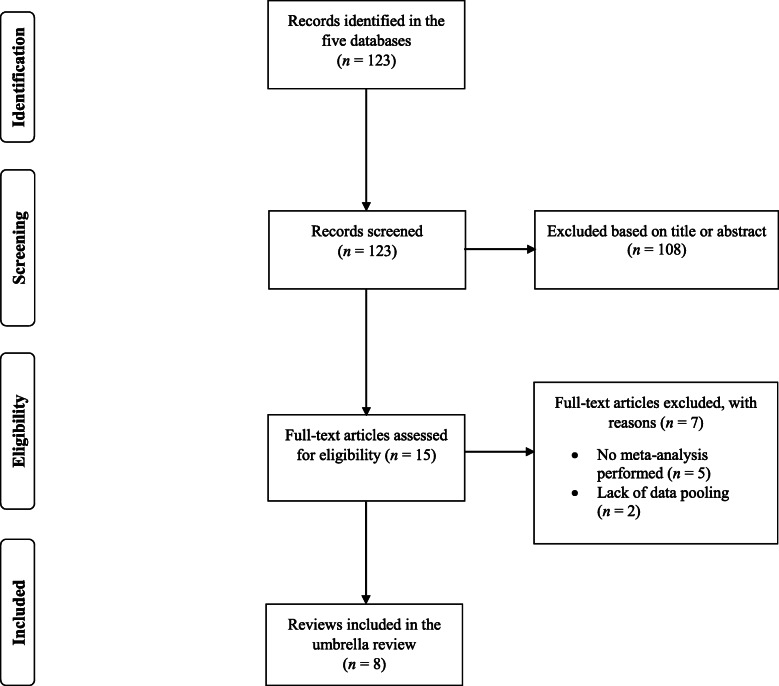
Flow-chart presenting the search process

In the five databases explored, the search syntax yielded a total of 123 results. Out of this number of search results, 15 full-text papers were read [13–20, 28–34], while other references were excluded based on their titles and/or abstracts. After reading the full-texts, seven reviews were excluded because they did not contain a meta-analysis [28–34]. Two of these reviews [31, 32] calculated effect sizes from the included primary studies but did not pool them using a meta-analysis, and, thus, they did not satisfy the inclusion criteria. Therefore, a total of eight meta-analyses [13–20] were included in the current umbrella review (Fig. 1).

### Summary of the included reviews

The number of studies included in each meta-analysis ranged from 5 to 26 (average: 13 studies; Table 1). The pooled number of participants per meta-analysis ranged from 46 to 241. The majority of participants in primary studies were males (77 to 100% of all participants included in the meta-analyses). The meta-analyses explored the effects of sodium bicarbonate supplementation on a range of exercise outcomes, including: general mean power (calculated as the change in performance in cycling, running, rowing or swimming tasks in high-intensity trials of short duration; 26 included studies), performance in endurance events lasting ∼45 s to 8 min (operationally defined as the time needed to complete an event of swimming, running, cycling or rowing; 25 included studies), muscle endurance (12 included studies), muscle strength (11 included studies), peak and mean power in single and repeated Wingate tests (3, 6, or 10 included studies), repeated-sprint performance (total work, best sprint and last sprint performance; 6 included studies), and Yo-Yo test performance (5 included studies).

**Table 1:** Summary of the meta-analyses included in the umbrella review

Reference	Included studies	Number of included primary studies (sample size)	Performance test/outcome	Effect size and ***p***-value	***I***^***2***^
***Meta-analyses that used Cohen’s d for data analysis***					
Christensen et al. [14]	Crossover study designs	25 studies (*n* = 235)	Endurance events lasting ∼45 s to 8 min	0.40 (95% CI: 0.27, 0.54); *p* < 0.001	n/a
Grgic [15]	Crossover study designs	10 studies (*n* = 102)	Mean and peak power in single and repeated Wingate tests	Peak power bout 1: − 0.01 (95% CI: − 0.06, 0.04); *p* = 0.730 Peak power bout 2: 0.02 (95% CI: − 0.10, 0.13); *p* = 0.774 Peak power bout 3: 0.09 (95% CI: 0.00, 0.17); *p* = 0.048 Peak power bout 4: 0.29 (95% CI: − 0.13, 0.71); *p* = 0.180 Mean power bout 1: 0.02 (95% CI: − 0.07, 0.11); *p* = 0.688 Mean power bout 2: 0.09 (95% CI: 0.03, 0.16); *p* = 0.005 Mean power bout 3: 0.21 (95% CI: − 0.16, 0.58); *p* = 0.268 Mean power bout 4: 0.62 (95% CI: 0.15, 1.08); *p* = 0.009	Peak power bout 1: 0% Peak power bout 2: 7% Peak power bout 3: 0% Peak power bout 4: 0% Mean power bout 1: 0% Mean power bout 2: 0% Mean power bout 3: 0% Mean power bout 4: 0%
Grgic et al. [16]	Crossover study designs	5 studies (*n* = 46)	Yo-Yo test performance	0.36 (95% CI: 0.10, 0.63); *p* = 0.007	14%
Grgic et al. [17]	Crossover study designs	13 studies for muscle endurance (*n* = 113)^a^ and 11 studies for muscle strength (*n* = 110)	Muscle endurance and muscle strength	Muscle endurance: 0.37 (95% CI: 0.15, 0.59); *p* = 0.001 Muscle strength: − 0.03 (95% CI: − 0.18, 0.12); *p* = 0.725	Muscle endurance: 70% Muscle strength: 45%
Lopes-Silva et al. [18]	Crossover study designs	6 studies (*n* = 77)	Measures of repeated-sprint ability (total work, best sprint, and last sprint performance)	Total work: 0.43 (95% CI: −0.11, 0.97); *p* = 0.12 Best sprint: 0.02 (95% CI: − 0.30, 0.34); *p* = 0.90 Last sprint: 0.20 (95% CI: − 0.13, 0.52); *p* = 0.14	Total work: 0% Best sprint: 0% Last sprint: 69%
Lopes-Silva et al. [19]	Crossover and between-group study designs	6 studies (*n* = 65) that used protocols of acute ingestion and 3 studies (*n* = 60) that used multi-day protocols of ingestion	Mean and peak power in single and repeated Wingate tests	*Acute ingestion* Peak power: 0.02 (95% CI: − 0.19, 0.23); *p* = 0.87 Mean power: 0.15 (95% CI: − 0.06, 0.36); *p* = 0.92 *Multi-day protocols of ingestion* Peak power: 1.21 (95% CI: 0.83, 1.42); *p* = 0.001 Mean power: 1.26 (95% CI: 0.96, 1.56); *p* = 0.001	*Acute ingestion* Peak power: 0% Mean power: 0% *Multi-day protocols of ingestion* Peak power: 27% Mean power: 88%
***Meta-analyses that used percent changes for data analysis***					
Carr et al. [13]	Crossover study designs	26 studies (*n* = 241)	General mean power	1.7% (90% CL: 90% CL: − 0.3%, 3.7)	n/a
Turnes et al. [20]	Crossover study designs	5 studies (*n* = 52)	Mean power in 2000-m rowing	1.4% (90% CL: 0.1, 2.6%)	n/a

*CI*: confidence interval; *CL*: confidence limit; ^a^12 studies were included in the meta-analysis

### Methodological quality and quality of evidence

The average score on the AMSTAR 2 checklist was 70% (range: 50 to 81%). Six reviews were categorized as being of moderate methodological quality, while two reviews were categorized as being of high quality (Table 2). Based on the GRADE checklist, the quality of evidence ranged from low to moderate. Low-quality evidence was found for general mean power, performance in endurance events lasting ∼45 s to 8 min, muscle endurance, mean and peak power output in the Wingate test (in one of two meta-analyses that explored this outcome), total work in repeated-sprints, and 2000-m rowing performance. Moderate quality evidence was found for muscle strength, peak and mean power in the Wingate test, best sprint and last sprint performance in repeated-sprints, and Yo-Yo test performance (Table 3).

**Table 2:** Result of the quality assessment using the Assessing the Methodological Quality of Systematic Reviews 2 (AMSTAR 2) checklist

Reference	AMSTAR 2 items																Score
	1	2	3	4	5	6	7	8	9	10	11	12	13	14	15	16	
Carr et al. [13]	Yes	No	Yes	Yes	Yes	Yes	No	Yes	No	No	Yes	n/a	n/a	Yes	Yes	Yes	63% MQ
Christensen et al. [14]	Yes	No	Yes	Yes	Yes	Unclear	No	Yes	Yes	Yes	Yes	Yes	Yes	Yes	No	Yes	75% MQ
Grgic [15]	Yes	No	Yes	Yes	No	No	No	Yes	Yes	No	Yes	Yes	Yes	Yes	Yes	Yes	69% MQ
Grgic et al. [16]	Yes	No	Yes	Yes	Yes	Yes	No	Yes	Yes	No	Yes	Yes	Yes	Yes	Yes	Yes	81% HQ
Grgic et al. [17]	Yes	No	Yes	Yes	Yes	Yes	No	Yes	Yes	No	Yes	Yes	Yes	Yes	Yes	Yes	81% HQ
Lopes-Silva et al. [18]	Yes	No	Yes	Yes	Yes	Unclear	No	Yes	Yes	No	Yes	Yes	Yes	Yes	Yes	Yes	75% MQ
Lopes-Silva et al. [19]	Yes	No	Yes	Yes	Yes	Unclear	No	Yes	Yes	No	Yes	Yes	Yes	Yes	No	Yes	69% MQ
Turnes et al. [20]	Yes	No	Yes	Yes	Unclear	Unclear	No	Yes	No	No	Yes	n/a	n/a	Yes	Yes	Yes	50% MQ

MQ: moderate quality; HQ: high quality

**Table 3:** Results of the quality of evidence assessment using the Grading of Recommendations Assessment, Development, and Evaluation (GRADE) criteria

Reference	GRADE items					Quality of the evidence*
	Risk of bias	Inconsistency	Indirectness	Imprecision	Publication bias	
Carr et al. [13]	Unclear (no quality assessment performed)	Not serious	Serious indirectness (only 15% of participants included in the analysis were women)	Not serious	Undetected	Low ⊕ ⊕ ΟΟ
Christensen et al. [14]	Not serious	Not serious	Serious indirectness (only 9% of participants included in the analysis were women)	Not serious	Strongly suspected (no “grey” literature searches; asymmetry of the funnel plot was not explored; the effect size of the largest study was smaller than the pooled estimate)	Low ⊕ ⊕ ΟΟ
Grgic [15]	Peak power: not serious	Peak power: not serious	Peak power: serious indirectness (none of the participants included in the analysis were women)	Peak power: not serious	Peak power: undetected	Peak power: moderate ⊕ ⊕ ⊕Ο
	Mean power: not serious	Mean power: not serious	Mean power: serious indirectness (only 15% of participants included in the analysis were women)	Mean power: not serious	Mean power: undetected	Mean power: moderate ⊕ ⊕ ⊕Ο
Grgic et al. [16]	Not serious	Not serious	Serious indirectness (none of the participants included in the analysis were women)	Not serious	Undetected	Moderate ⊕ ⊕ ⊕Ο
Grgic et al. [17]	Muscle endurance: not serious	Not serious	Serious indirectness (only 5% of participants included in the analysis were women)	Not serious	Strongly suspected (“grey” literature searches were performed; however, asymmetry of the funnel plot was not explored and the effect size of the largest study was smaller than the pooled estimate)	Low ⊕ ⊕ ΟΟ
	Muscle strength: not serious	Not serious	Serious indirectness (only 4% of participants included in the analysis were women)	Not serious	Undetected	Moderate ⊕ ⊕ ⊕Ο
Lopes-Silva et al. [18]	Total work: not serious	Total work: not serious	Total work: serious indirectness (only 28% of participants included in the analysis were women)	Total work: serious limitation	Total work: undetected	Low ⊕ ⊕ ΟΟ
	Best sprint: not serious	Best sprint: not serious	Best sprint: serious indirectness (only 23% of participants included in the analysis were women)	Best sprint: not serious	Best sprint: undetected	Moderate ⊕ ⊕ ⊕Ο
	Last sprint: not serious	Last sprint: not serious	Last sprint: serious indirectness (only 23% of participants included in the analysis were women)	Last sprint: not serious	Last sprint: undetected	Moderate ⊕ ⊕ ⊕Ο
Lopes-Silva et al. [19]	Acute ingestion, peak power: not serious	Acute ingestion, peak power: not serious	Acute ingestion, peak power: serious indirectness (none of the participants included in the analysis were women)	Acute ingestion, peak power: not serious	Acute ingestion, peak power: strongly suspected (no “grey” literature searches; asymmetry of the funnel plot was not explored; the effect size of the largest study was similar to the pooled estimate)	Low ⊕ ⊕ ΟΟ
	Acute ingestion, mean power: not serious	Acute ingestion, mean power: not serious	Acute ingestion, mean power: serious indirectness (none of the participants included in the analysis were women)	Acute ingestion, mean power: not serious	Acute ingestion, mean power: strongly suspected (no “grey” literature searches; asymmetry of the funnel plot was not explored; the effect size of the largest study was similar to the pooled estimate)	Low ⊕ ⊕ ΟΟ
	Multi-day ingestion, peak power: not serious	Multi-day ingestion, peak power: not serious	Multi-day ingestion, peak power: serious indirectness (none of the participants included in the analysis were women)	Multi-day ingestion, peak power: not serious	Multi-day ingestion, peak power: strongly suspected (no “grey” literature searches; asymmetry of the funnel plot was not explored; the effect size of the largest study was similar to the pooled estimate)	Low ⊕ ⊕ ΟΟ
	Multi-day ingestion, mean power: not serious	Multi-day ingestion, mean power: not serious	Multi-day ingestion, mean power: serious indirectness (none of the participants included in the analysis were women)	Multi-day ingestion, mean power: not serious	Multi-day ingestion, mean power: strongly suspected (no “grey” literature searches; asymmetry of the funnel plot was not explored; the effect size of the largest study was similar to the pooled estimate)	Low ⊕ ⊕ ΟΟ
Turnes et al. [20]	Unclear (no quality assessment performed)	Not serious	Serious indirectness (only 10% of participants included in the analysis were women)	Not serious	Undetected	Low ⊕ ⊕ ΟΟ

Studies were classified as: ⊕ ⊕ ⊕ ⊕ = high quality; ⊕ ⊕ ⊕Ο = moderate quality; ⊕ ⊕ ΟΟ = low quality; ⊕ΟΟΟ = very low quality

### Effects of sodium bicarbonate supplementation on exercise performance

#### Meta-analyses that used Cohen’s *d*

**Fig. 2:**
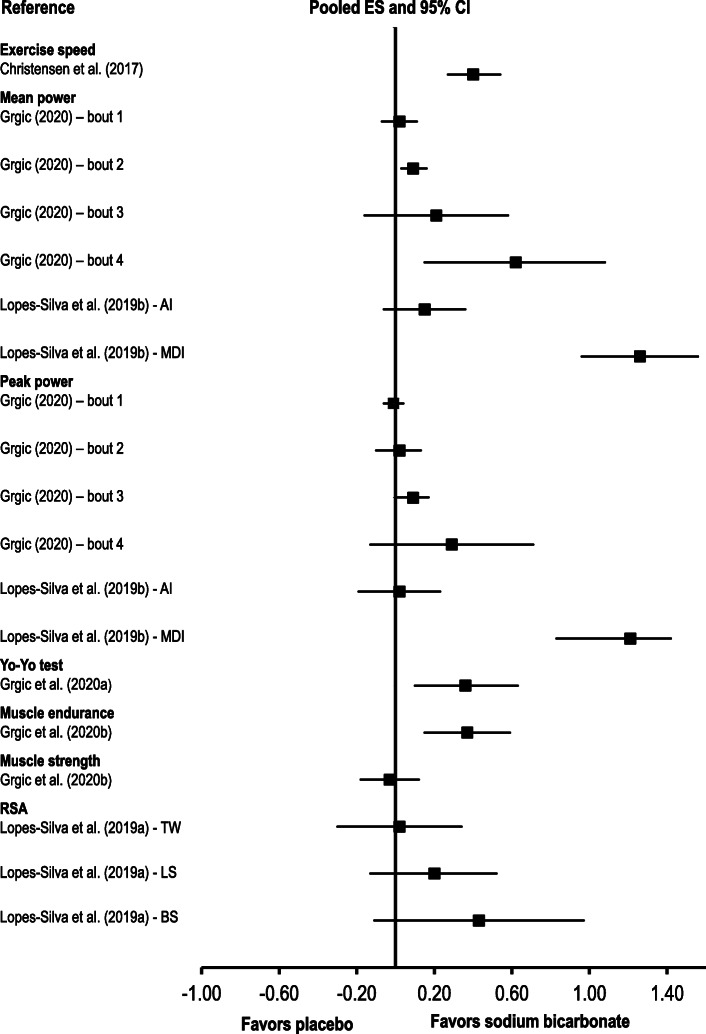
Summary of pooled effect sizes (ES) and 95% confidence intervals (CI) from the meta-analyses that used Cohen’s *d* for data analysis. AI: acute ingestion of sodium bicarbonate; BS: best sprint; LS: last sprint; MDI: multi-day ingestion of sodium bicarbonate; RSA: repeated-sprint ability; TW: total work

A meta-analysis of 25 studies reported ergogenic effects of sodium bicarbonate supplementation on performance in endurance events lasting ∼45 s to 8 min (pooled effect size: 0.40; Fig. 2; Table 4) [14].

**Table 4:** Effects of sodium bicarbonate supplementation on exercise performance: summary findings, methodological quality of literature reviews, and quality of evidence

Quality of evidence	Methodological quality of literature reviews	
	Moderate	High
*Meta-analyses that found significant ergogenic effects of sodium bicarbonate*		
Low	• Endurance events lasting ∼45 s to 8 min in Christensen et al. [14] • Anaerobic power in the Wingate test using the multiple-day supplementation protocol in Lopes-Silva et al. [19] • 2000-m rowing in Turnes et al. [20]	• Muscle endurance in Grgic et al. [17]
Moderate	• Anaerobic power in the repeated-bout Wingate test in Grgic [15]	• Yo-Yo test performance in Grgic et al. [16]
*Meta-analyses that did not find significant ergogenic effects of sodium bicarbonate*		
Low	• General mean power in Carr et al. [13] • Repeated-sprint ability (total work) in Lopes-Silva et al. [18] • Anaerobic power in the Wingate test using single-dose supplementation protocol in Lopes-Silva et al. [19]	/
Moderate	• Repeated-sprint ability (best sprint) in Lopes-Silva et al. [18] • Repeated-sprint ability (last sprint) in Lopes-Silva et al. [18]	• Muscle strength in Grgic et al. [17]

Note: Quality of evidence was evaluated using the GRADE criteria; methodological quality of the review was evaluated using the AMSTAR 2 checklist

In a meta-analysis including data from 12 studies, sodium bicarbonate supplementation was found to be ergogenic for muscular endurance (pooled effect size: 0.37) [17]. In a meta-analysis including 11 studies, no ergogenic effect was observed for muscular strength (pooled effect size: − 0.03) [17].

Two meta-analyses examined the effects of acute sodium bicarbonate ingestion on mean and peak power in the single and repeated Wingate tests [15, 19]. In a meta-analysis that included six studies, there were no ergogenic effects of acute sodium bicarbonate ingestion on mean and peak power in Wingate bout 1, 2 and 3 (pooled effect size range: − 0.07 to 0.22) [19]. In another meta-analysis with 10 included studies [15], an ergogenic effect of sodium bicarbonate supplementation was found on mean power in Wingate bout 2 (pooled effect size: 0.09) and bout 4 (pooled effect size: 0.62), as well as peak power in Wingate bout 3 (pooled effect size: 0.09). No significant differences between the effects of sodium bicarbonate and placebo were observed in other comparisons (i.e., mean power in Wingate bout 1 and 3 and peak power in Wingate bout 1, 2 and 4).

The effects of multi-day protocols of sodium bicarbonate ingestion on mean and peak power in single and repeated Wingate tests were examined in one meta-analysis that included 3 studies [19]. Sodium bicarbonate ingestion was ergogenic for peak and mean power (pooled effect size range: 1.21 to 1.26).

The effects of sodium bicarbonate supplementation on repeated-sprint performance measures were explored in one meta-analysis that included 6 studies [18]. No significant difference between the effects of sodium bicarbonate and placebo was found for any of the analyzed outcomes (pooled effect size range: 0.02 to 0.43).

One meta-analysis, including 5 studies, examined the effects of sodium bicarbonate supplementation on Yo-Yo test performance and reported an ergogenic effect of sodium bicarbonate (pooled effect size: 0.36) [16].

#### Meta-analyses that used percent changes

**Fig. 3:**
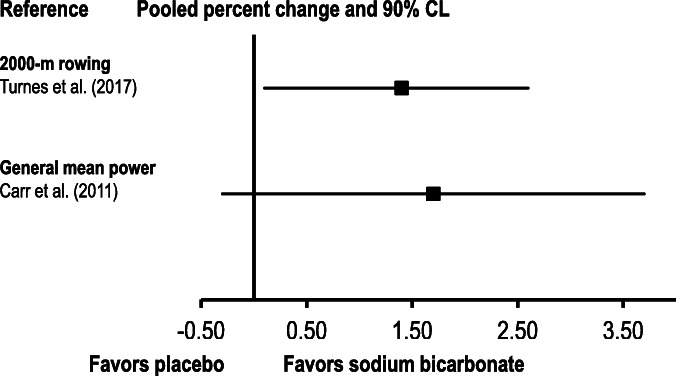
Summary of pooled percent changes and 90% confidence limits (CL) from the meta-analyses that used percent changes for data analysis

In one meta-analysis [13], exercise performance data from 25 included studies were converted to general mean power. In this analysis, there was no significant difference between sodium bicarbonate and placebo (1.7%; 90% confidence limit [CL]: − 0.3, 3.7%; Fig. 3).

One meta-analysis included 6 studies on the effects of sodium bicarbonate supplementation on 2000-m rowing performance [20]. Sodium bicarbonate supplementation was found to enhance this outcome by 1.4% (90% CL: 0.1, 2.6%).

#### Subgroup analyses

Besides the main analyses, three reviews [13, 17, 19] also conducted additional subgroup meta-analyses (Table 5). In the meta-analysis [13] with general mean power as the outcome variable, subgroup analyses included sodium bicarbonate dose, number of exercise bouts, exercise test duration, participants’ sex and training status, and non-blinded (blinding of participants) study designs. A significant ergogenic effect of sodium bicarbonate (0.6%; 90% CL: 0.2, 1.0%) was found only when five extra exercise bouts were performed.

**Table 5:** Findings of subgroup analyses reported in the included reviews

Reference	Subgroup analyses focus	Subgroups analyses results
Carr et al. [13]	Increase in dose by 1 mmoL/kg/body mass	0.5% (90% CL: −0.1, 0.6%)
	Five extra bouts	0.6% (90% CL: 0.2, 1.0%)
	10 × duration	− 0.6% (90% CL: − 1.2, 0.3%)
	Non-athletes	−1.1% (90% CL: − 2.2, 0.0%)
	Females	−0.7% (90% CL: − 2.1, 0.7%)
	Non-blinded	0.2% (90% CL: −0.5, 0.9%)
Grgic et al. [17] – muscle endurance	Large muscle groups	ES: 0.40 (95% CI: 0.13, 0.66)
	Small muscle groups	ES: 0.31 (95% CI: 0.04, 0.59)
	One time point of ingestion	ES: 0.53 (95% CI: 0.14, 0.93)
	Multiple time points of ingestion	ES: 0.23 (95% CI: 0.05, 0.42)
Grgic et al. [17] – muscle strength	Tested in a rested state	ES: 0.02 (95% CI: − 0.09, 0.13)
	Tested in a fatigued state	ES: −0.16 (95% CI: − 0.59, 0.28)
	One time point of ingestion	ES: −0.14 (95% CI: − 0.50, 0.21)
	Multiple time points of ingestion	ES: 0.04 (95% CI: −0.06, 0.14)
Lopes-Silva et al. [19] – peak power	Acute ingestion: Wingate bout 1	ES: − 0.07 (95% CI: − 0.36, 0.23)
	Acute ingestion: Wingate bout 2	ES: 0.00 (95% CI: − 0.42, 0.42)
	Acute ingestion: Wingate bout 3	ES: 0.14 (95% CI: −0.28, 0.56)
	Multi-day ingestion: Wingate bout 1	ES: 0.79 (95% CI: 0.20, 1.37)
	Multi-day ingestion: Wingate bout 2	ES: 1.52 (95% CI: 0.90, 2.13)
	Multi-day ingestion: Wingate bout 3	ES: 0.89 (95% CI: 0.33, 1.45)
	Multi-day ingestion: Wingate bout 4	ES: 1.36 (95% CI: 0.76, 1.96)
Lopes-Silva et al. [19] – mean power	Acute ingestion: Wingate bout 1	ES: 0.12 (95% CI: −0.18, 0.41)
	Acute ingestion: Wingate bout 2	ES: 0.14 (95% CI: −0.28, 0.56)
	Acute ingestion: Wingate bout 3	ES: 0.22 (95% CI: −0.20, 0.65)
	Multi-day ingestion: Wingate bout 1	ES: 0.91 (95% CI: 0.43, 1.39)
	Multi-day ingestion: Wingate bout 2	ES: 1.04 (95% CI: 0.46, 1.62)
	Multi-day ingestion: Wingate bout 3	ES: 1.66 (95% CI: 0.95, 2.38)
	Multi-day ingestion: Wingate bout 4	ES: 2.09 (95% CI: 1.31, 2.87)

ES: effect size; CL: confidence limit; CI: confidence interval

In the review with muscle endurance and muscle strength as outcome variables [17], subgroup meta-analyses included the size of the exercised muscle, protocol of ingestion, and testing in a fatigued vs. non-fatigued state. The results of these subgroup meta-analyses were consistent with those reported in the primary meta-analyses, confirming an ergogenic effect of sodium bicarbonate on muscle endurance and finding no significant difference between the effects of sodium bicarbonate and placebo on muscle strength (Table 5).

Finally, Lopes-Silva et al. [19] performed subgroup analyses to explore the effects of single-dose and multi-day protocols of sodium bicarbonate ingestion on peak and mean power in Wingate bouts 1–4. As with the primary findings, subgroup analyses found that multi-day protocol of supplementation increased peak and mean power in bouts 1–4. No significant difference was found between the effects of placebo and acute sodium bicarbonate ingestion.

## Discussion

### Main findings of the review

Based on the meta-analytic evidence, it can be concluded that sodium bicarbonate supplementation acutely enhances peak anaerobic power, anaerobic capacity, performance in endurance events lasting ∼45 s to 8 min, muscle endurance, 2000-m rowing performance, and high-intensity intermittent running. This conclusion is based on reviews of moderate and high methodological quality. Moderate quality evidence was found for the ergogenic effects of sodium bicarbonate supplementation on peak and mean power in the Wingate test and Yo-Yo test performance. Low quality evidence was found for the ergogenic effect of sodium bicarbonate supplementation on performance in endurance events lasting ∼45 s to 8 min, muscle endurance, and 2000-rowing performance. The ergogenic effects ranged from trivial (pooled effect size: 0.09) to large (pooled effect size: 1.26). Still, for most outcomes, sodium bicarbonate elicited comparable ergogenic effects. For example, sodium bicarbonate produced similar effects on performance in endurance events lasting ∼45 s to 8 min, muscle endurance tests, and Yo-Yo test (pooled effect size range: 0.36 to 0.40). No significant difference between the effects of sodium bicarbonate and placebo was found for general mean power, muscle strength and repeated-sprint ability.

### Generalizability of the results

Most primary studies included in the meta-analyses were conducted among male participants, which limits the generalizability of findings. Specifically, 77 to 100% of participants included in the meta-analyses were males. Due to the uneven distribution of sexes in primary studies, all included reviews were categorized as having “serious indirectness” in the GRADE assessment [27]. One included review performed a subgroup analysis that only considered findings among females (pooled sample size *n* = 36) [13]. In this subgroup analysis, the pooled effect of sodium bicarbonate supplementation on general mean power was − 0.7% (90% confidence limit: − 2.1, 0.7%) and was concluded to be “unclear”. However, it should be mentioned that no significant difference between sodium bicarbonate and placebo for general mean power was found in the main meta-analysis of this review (that included both females and males). While a handful of studies [35–38] conducted in females reported ergogenic effects of sodium bicarbonate on exercise performance, additional research in this population is needed to draw stronger inferences. Future studies should consider including both men and women and analyze their data separately to determine if there is a difference in responses to sodium bicarbonate supplementation between sexes. It should be noted, however, that such comparisons would require larger sample sizes to ensure adequate statistical power.

### Effects of sodium bicarbonate supplementation on exercise performance

When ergogenic, it seems that the effectiveness of sodium bicarbonate supplementation is similar for different exercise tasks. Sodium bicarbonate was comparably ergogenic for performance in endurance events lasting ∼45 s to 8 min, muscle endurance, and Yo-Yo test performance (pooled effect size range: 0.36 to 0.40) [14, 16, 17]. Therefore, it seems that small-to-moderate effects of sodium bicarbonate supplementation on exercise performance may be expected, which may be practically meaningful in sports training and competition. While this was not the main topic of this review, a brief mention of the mechanisms that explain these ergogenic effects is also needed. During intense exercise, the accumulation of H^+^ and decrease in pH may contribute to fatigue and decreased performance due to its effects on glycolytic enzymes, Ca^2+^ sensitivity, and cross-bridge cycling [39, 40]. Sodium bicarbonate ingestion may help to delay exercise-induced fatigue and improve performance, as it acts by increasing H^+^ efflux and improving intramuscular acid-base (for a detailed review on the mechanisms, see [41]).

Most primary studies on this topic employed acute sodium bicarbonate supplementation protocols (e.g., a single dose consumed 3 h before exercise). However, one meta-analysis reported large effects among studies that used multi-day protocols of sodium bicarbonate supplementation [19]. Specifically, this meta-analysis included studies that provided daily sodium bicarbonate supplementation for 5 to 7 days before the exercise test (e.g., 4 × 125 mg per day), with additional sodium bicarbonate ingestion a few hours before the test. The advantage of this protocol is that it may reduce sodium bicarbonate-induced side effects, given that smaller doses are ingested throughout the day [41]. In this analysis, the effect size of sodium bicarbonate supplementation on peak and mean power recorded during single and repeated Wingate tests ranged from 0.79 to 2.03. The effects of acute sodium bicarbonate supplementation (i.e., only a single dose consumed 90 to 180 min before exercise) on Wingate test performance were smaller (pooled effect size range: 0.09 to 0.62). This would suggest that greater ergogenic effects may be observed when using multi-day protocols of sodium bicarbonate supplementation. However, these findings were based on only three studies [42–44], none of which directly compared the utilized protocols of supplementation to protocols of acute sodium bicarbonate ingestion. Therefore, further research is needed to compare the effects of acute vs. multi-day protocols of sodium bicarbonate supplementation on different exercise tasks and in different populations. One study [45] explored the effects of both protocols and reported that they have similar ergogenic effects on repeated-sprint ability. Another study [46] compared the effects of single-dose and multi-day protocols of sodium bicarbonate ingestion on cycling performance on three consecutive testing days. While there was no significant difference between the protocols on the first testing day, a greater performance-enhancing effect of multi-day protocol was found on the second and third testing days. Due to the paucity of research, future work on this topic is needed. Future work is also needed to explore the effects of long-term sodium bicarbonate supplementation on different exercise performance outcomes, given that only a handful of studies [47–50] have explored this thus far.

Meta-analyses are commonly used to overcome the limitations of small sample sizes in primary studies. However, whether this is achieved or not depends on the pooled sample size. Given their relatively small sizes of pooled sample, some of the analyses included in this review might not have provided definitive answers regarding the effects of sodium bicarbonate supplementation on exercise performance. Specifically, one meta-analysis did not find significant differences between the effects of sodium bicarbonate and placebo in three measures of repeated-sprint performance [18]. In the analysis for total work, the effects favored sodium bicarbonate (pooled effect size: 0.43; 95% CI: − 0.11, 0.97), but the difference compared to placebo was not statistically significant (*p* = 0.12). The lack of statistical significance in this analysis could be attributed to the fact that only three studies with a relatively small pooled sample size (*n* = 27) were included, hence making results susceptible to type II error. Therefore, non-significant results of this analysis might not necessarily reflect the absence of an effect in the population. Due to the limited number of primary studies, future research should explore the effects of sodium bicarbonate supplementation on different measures of repeated-sprint performance.

### Methodological quality

All included reviews were classified as being of moderate or high quality. Therefore, the findings presented in this umbrella review are not confounded by low methodological quality of included reviews. Nevertheless, there are some limitations noted on the AMSTAR 2 checklist that should be considered for future reviews on the topic. For example, none of the included reviews received a point on item 7, which refers to reporting of excluded studies. Future reviews should consider adding a list of excluded studies and provide reasons for their exclusion. This will make the results of the study selection process more transparent and easier to verify. Only one review [14] reported funding sources for the included studies and received a point on item 10. The review noted that none of the included primary studies received funding from sources that might have had a potential commercial interest. Future reviews and primary studies should present information on received funding, as it has been shown that findings of nutrition-related research may be biased in favor of sponsors’ products [51].

## Conclusion

Based on meta-analyses of moderate to high quality, it can be concluded that sodium bicarbonate supplementation acutely enhances peak anaerobic power, anaerobic capacity, performance in endurance events lasting ∼45 s to 8 min, muscle endurance, 2000-m rowing performance, and high-intensity intermittent running. The ergogenic effects ranged from trivial (pooled effect size: 0.09) to large (pooled effect size: 1.26). Still, for most outcomes, sodium bicarbonate elicited comparable ergogenic effects. For example, sodium bicarbonate produced similar effects on performance in endurance events lasting ∼45 s to 8 min, muscle endurance tests, and Yo-Yo test (pooled effect size range: 0.36 to 0.40). The quality of evidence presented in the included meta-analyses ranged from low to moderate. More research is needed among women to improve the generalizability of findings.

## Data Availability

Not applicable.

## Abbreviations

AMSTAR 2: Assessing the Methodological Quality of Systematic Reviews 2
CI: Confidence Interval
GRADE: Grading of Recommendations Assessment, Development, and Evaluation
IOC: International Olympic Committee
PICO: Participant-Intervention-Comparison-Outcome
